# Supplementary material for: European polygenic risk score for prediction of breast cancer shows similar performance in Asian women
Source: Nat Commun. 2020 Jul 31;11:3833. doi: 10.1038/s41467-020-17680-w (PMC7395776; doi:10.1038/s41467-020-17680-w)
Supplement: Supplementary file 1 — Supplementary Information [file 41467_2020_17680_MOESM1_ESM.pdf]

Supplementary information

European polygenic risk score for prediction of breast cancer shows similar performance in Asian women

Ho et al.

Supplementary Figure 1. Principal components analysis

Principal components analysis of population structure of Malaysian and Singaporean Chinese, Malay, and Indian from Malaysian Breast Cancer Genetics (MyBrCa) batch 2 and Singapore Breast Cancer Cohort (SGBCC) batch 2 studies. First two principal components (PCs) are shown here. Each individual is represented by one dot and the colour label corresponding to their self-reported ethnic origin.

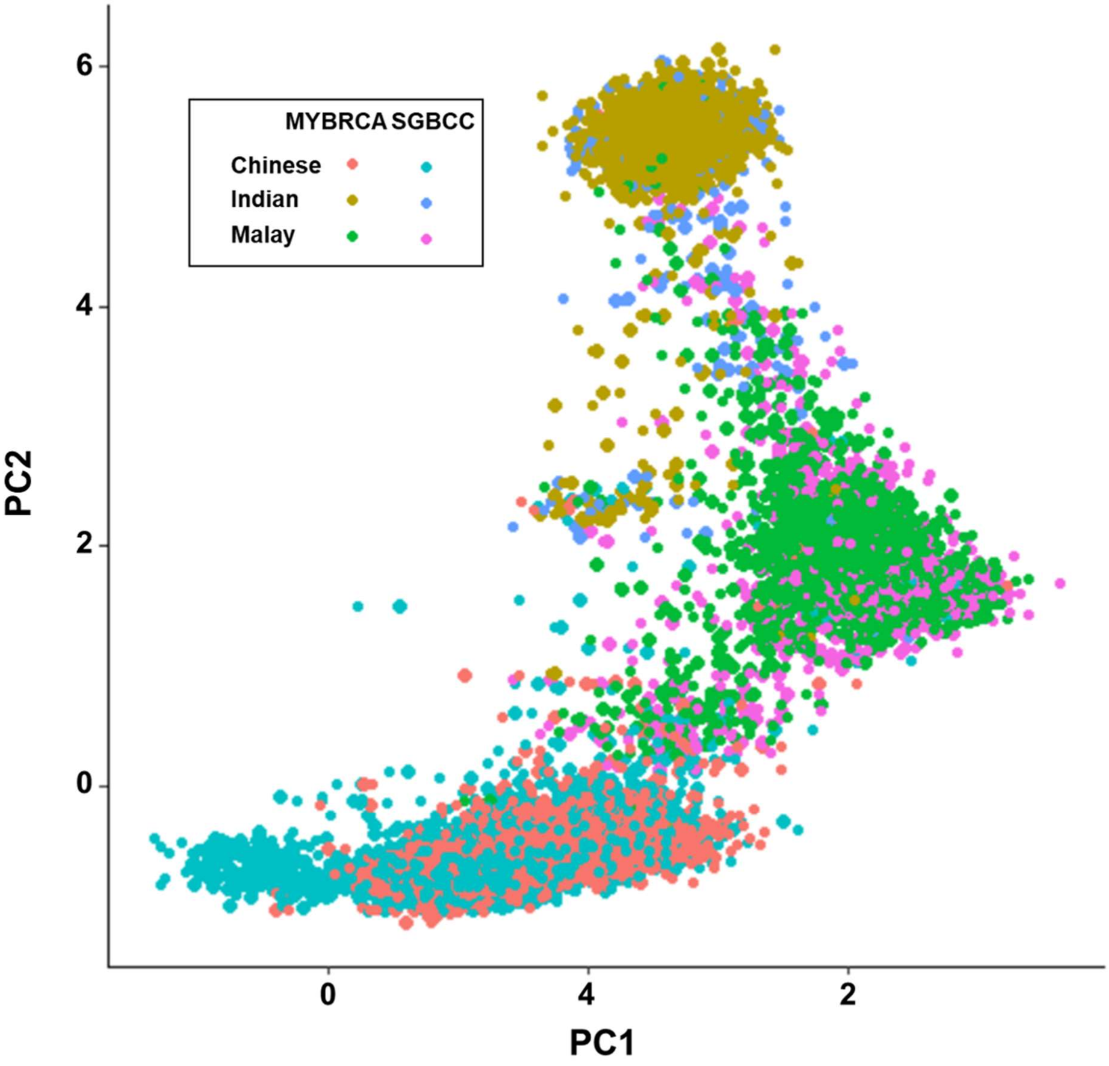

Supplementary Figure 2. Absolute risk using Singaporean breast cancer incidence. Lifetime (top panel) and 10-year absolute risk (bottom panel) of developing breast cancer for Chinese, Malay and Indian women calculated using Singaporean incidence and mortality data and using PRS relative risks for overall breast cancer. The solid horizontal black line shows the 2.3% risk threshold corresponds to the average 10-year risk of breast cancer for women of European ancestry at 50 years old. The analysis was conducted on 10,392 Chinese, 2,416 Malays and 1,598 Indians. Source data can be found in Table 5.

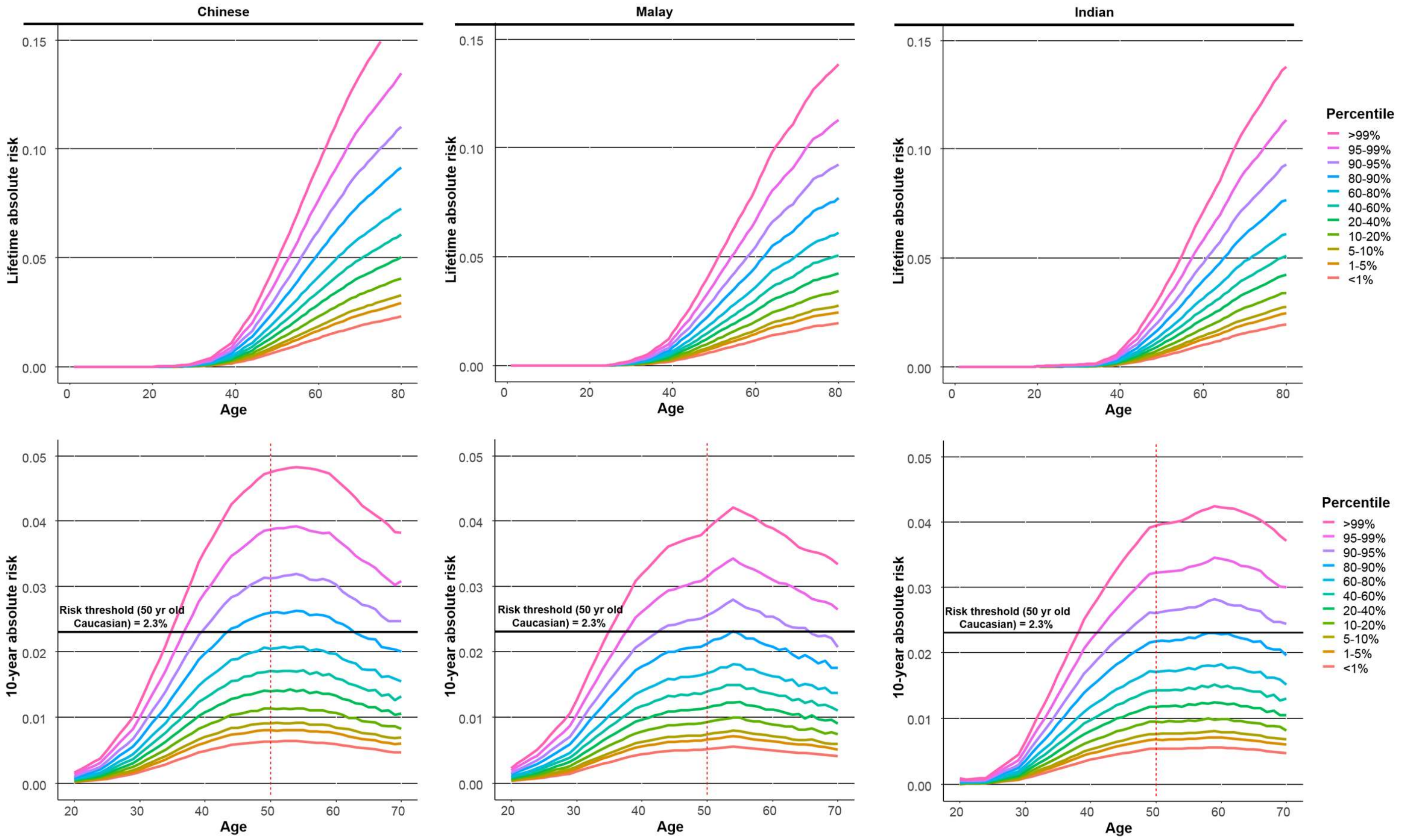

Supplementary Figure 3. Proportion of cases accounted for in the fraction of population above a specific 10-year risk threshold. The proportion of women in the general population (solid line) who would have maximum 10-year absolute risk of breast cancer (between age 20-70), adjusting for competing mortality, above a specific risk threshold and the proportion of cases (dashed lines) captured for 10,392 Chinese, 2,416 Malays and 1,598 Indians. The vertical grey dotted line (2.3%) is the average 10-year absolute risk of breast cancer of a 50-year old European ancestry woman. For Chinese women, about 25% of the population (16% for Malays and 17% for Indians) would reach the 2.3% risk threshold at some point in their lives and ~40% (27% for Malays and 28% for Indians) of all breast cancer cases occur in this 25% (16% for Malays and 17% for Indians) of the population (horizontal grey dotted lines). The plot was created using the estimated OR per SD (1.48) of PRS with overall breast cancer risk and ethnic-specific breast cancer incidences and mortality of Singaporean women.

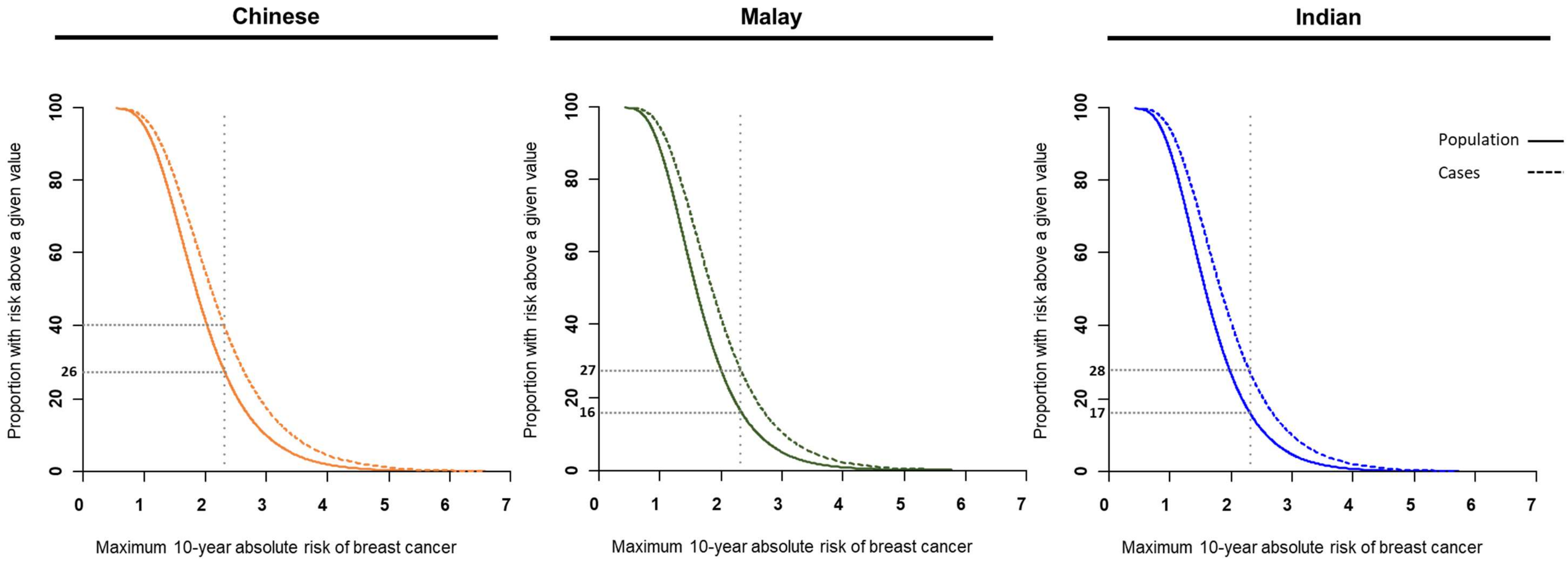

Supplementary Figure 4. Absolute risk using Australian breast cancer incidence. (a) Lifetime and (b) 10-year absolute risk of developing breast cancer using Australian incidence and mortality data and using PRS relative risks for overall breast cancer. The solid horizontal black line shows the 2.3% risk threshold corresponds to the average 10-year risk of breast cancer

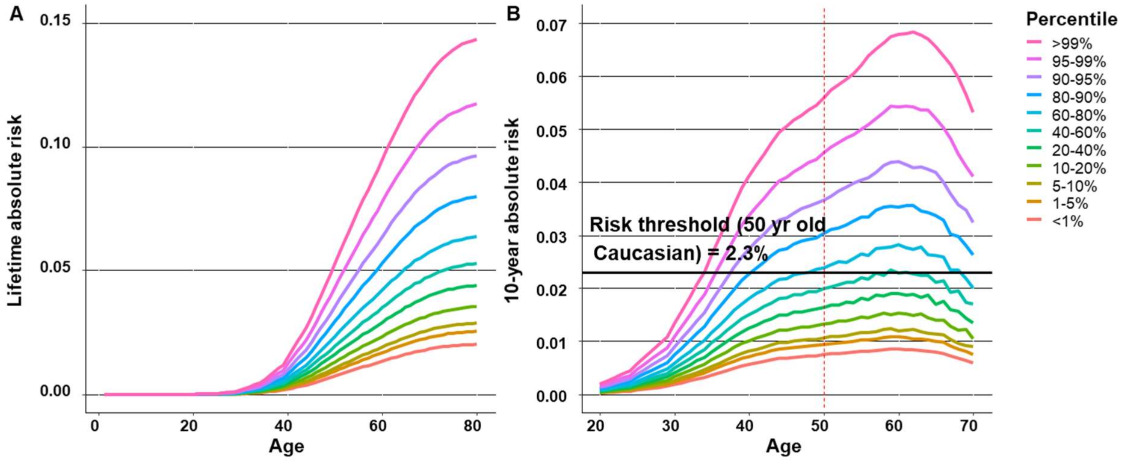

Supplementary Table 1. Participating studies and the number of individuals used in polygenic risk scores evaluation analyses.

Array type	Study acronym	Study Name	Study design	Country	Total		Mean age† (SD)		Cases ER status			Control family history			Cases family history		
					Control	Cases	Control	Cases	ER+	ER-	Un-known	Yes	No	Un-known	Yes	No	Un-known
**Case-control studies in BCAC Asians**																	
iCOGS	**ACP**	Asia Cancer Program	Hospital based case-control study	Thailand	636	416	46.30 (10.45)	47.08 (9.30)	89	52	275	11	625	0	19	397	0
	**HERPACC**	Hospital-based Epidemiologic Research Program at Aichi Cancer Center[1]	Hospital-based case-control study	Japan	1376	561	51.39 (10.93)	51.82 (11.15)	344	124	93	70	1190	116	42	480	39
	**SBCGS**	Shanghai Breast Cancer Genetic Study[2]	Population-based case-control study, cohort study	China	892	829	53.02 (9.73)	54.63 (10.05)	498	271	60	19	873	0	45	784	0
	**SEBCS**	Seoul Breast Cancer Study[3,4]	Hospital-based case-control study	Korea	1129	1027	52.23 (7.73)	48.61 (8.98)	618	369	40	4	1	1124	35	472	520
	**TWBCS**	Taiwanese Breast Cancer Study[5,6]	Hospital-based case-control study	Taiwan	236	776	51.14 (7.85)	51.67 (11.25)	398	179	199	20	216	0	65	700	11
Oncoarray	**MyBrCa Batch 1**	Malaysian Breast Cancer Genetic Study[7]	Hospital-based case-control study	Malaysia	1254	797	52.52 (8.08)	51.10 (10.84)	503	279	15	152	1102	0	114	683	0
	**MyBrCa Batch 2**	Malaysian Breast Cancer Genetic Study[7]	Hospital-based case-control study	Malaysia	2205	2509	54.9 (8.4)	51.57 (11.12)	1649	754	106	271	1934	0	324	2185	0
	**SGBCC Batch 1**	Singapore Breast Cancer Cohort	Hospital based breast cancer cohort and population based controls	Singapore	704	711	51.38 (10.14)	52.99 (10.78)	501	187	23	24	263	417	96	595	20
	**SGBCC Batch 2**	Singapore Breast Cancer Cohort	Hospital based breast cancer cohort and population based controls	Singapore	3343	2883	49.96 (9.94)	53.79 (10.49)	2063	665	155	0	0	3343	458	2357	68
	**ACP**	Asia Cancer Program	Hospital based case-control study	Thailand	642	448	41.35 (11.01)	49.38 (8.87)	273	144	31	15	411	216	28	387	33
	**HERPACC**	Hospital-based Epidemiologic Research Program at Aichi Cancer Center[1]	Hospital-based case-control study	Japan	283	231	52.17 (10.70)	52.08 (10.82)	192	38	1	14	243	26	21	195	15
	**HKBCS**	Hong Kong Breast Cancer Study[8,9]	Hospital-based case-control study	China	454	478	48.08 (10.40)	43.86 (9.97)	307	154	17	47	399	8	105	373	0
	**KOHBRA**	Korean Hereditary Breast Cancer Study[10]	Population-based Case-Control study	Korea	665	1292	44.69 (10.54)	39.98 (9.50)	761	427	104	99	565	1	262	1007	23
	**NGOBCS**	Nagano Breast Cancer Study[11]	Hospital-based case-control study	Japan	366	366	53.83 (9.96)	53.72 (10.61)	277	89	0	22	340	4	26	329	11
	**SBCGS**	Shanghai Breast Cancer Genetic Study[2]	Population-based case-control study, cohort study	China	935	815	57.05 (7.11)	55.29 (9.05)	491	289	35	22	913	0	31	784	0
	**SEBCS**	Seoul Breast Cancer Study[3,4]	Hospital-based case-control study	Korea	1107	1102	49.15 (9.69)	48.70 (9.71)	669	433	0	10	277	820	0	0	1102
	**TWBCS**	Taiwanese Breast Cancer Study[5,6]	Hospital-based case-control study	Taiwan	256	514	45.73 (10.10)	53.09 (11.41)	356	157	1	31	225	0	0	14	500
		**Total**			**16483**	**15755**			**9989**	**4611**	**1155**	**831**	**9577**	**6075**	**1671**	**11742**	**2342**
**Case-control studies for Asian within American studies**																	
iCOGS	**LAABC**	Los Angeles County Asian-American Breast Cancer Case-Control Study[12]	Population-based case-control study	USA	990	808	53.77 (10.17)	53.91 (10.68)	527	138	143	0	0	990	0	0	808
Oncoarray	**NC-BCFR**	Northern California Breast Cancer Family Registry[13]	Population-based familial case-control study	USA	52	446	45.19 (7.75)	48.29 (9.24)	285	109	52	6	46	0	141	305	0
	**CBCS**	Canadian Breast Cancer Study[14-17]	Population-based case-control study	Canada	170	253	54.41 (9.69)	52.98 (11.11)	210	33	10	15	150	5	28	213	12
		**Total**			**1212**	**1507**			**1022**	**280**	**205**	**21**	**196**	**995**	**169**	**518**	**820**
**Prospective cohort**																	
**GSA***	**SCHS**	Singapore Chinese Health Study[18,19]	Population-based prospective cohort	Singapore	9842	413	54.48 (2.55)	53.76 (2.34)	-	-	-	-	-	-	-	-	-

BCAC – Breast Cancer Association Consortium; *GSA - Global Screening Array; †Age of diagnosis for cases and age of consent for controls

Supplementary Table 2. Interaction between standardized PRSs and age or family history for overall and subtype-specific breast cancer

	Overall breast cancer		ER positive		ER negative	
	**OR (95% CI)**	**p-value**	**OR (95% CI)**	**p-value**	**OR (95% CI)**	**p-value**
**Model: PRS+age+PRS*age**						
Interaction between PRS and age	1.00 (0.998 - 1.00)	0.736	1.00 (0.996 - 1.000)	0.383	1.00 (0.996 - 1.00)	0.981
**Model: PRS+FH+PRS*FH**						
Interaction between PRS and family history	1.10 (0.98 - 1.23)	0.101	1.01 (0.97 - 1.25)	0.141	1.05 (0.90 - 1.23)	0.528

Supplementary Table 3. Association between standardised 287-SNP PRS and overall breast cancer risk by age categories

Age Group (years)	OR per SD (95% CI)
<40	1.47 (1.35-1.59)
40-50	1.54 (1.47-1.61)
50-60	1.53 (1.46-1.60)
60-70	1.55 (1.45-1.65)
70-80	1.50 (1.31-1.73)

Association between standardised 287-SNP polygenic risk scores (PRS) and overall breast cancer risk in different age categories.

Supplementary Table 4. Association between standardised 287-SNP polygenic risk scores (PRS) and breast cancer risk in Asian studies: observed and predicted odds ratio by PRS percentiles

Percentile (%)	Overall breast cancer				ER-positive disease				ER-negative disease			
	Case, N	Control, N	Estimated OR (95% CI)	Predicted OR	Case, N	Control, N	Estimated OR (95% CI)	Predicted OR	Case, N	Control, N	Estimated OR (95% CI)	Predicted OR
<1	52	165	0.38(0.27-0.52)	0.33	22	165	0.25 (0.16-0.39)	0.29	22	165	0.57 (0.36-0.90)	0.38
1-5	271	660	0.48 (0.41-0.56)	0.45	146	660	0.40 (0.33-0.49)	0.41	101	660	0.62 (0.50-0.78)	0.51
5-10	379	824	0.54 (0.48-0.62)	0.54	208	824	0.46 (0.39-0.54)	0.51	147	824	0.71 (0.59-0.79)	0.6
10-20	962	1,648	0.67 (0.61-0.74)	0.65	575	1,648	0.64 (0.57-0.71)	0.61	283	1,648	0.68 (0.59-0.90)	0.68
20-40	2376	3,297	0.83 (0.77-0.90)	0.8	1,447	3,297	0.79 (0.73-0.86)	0.79	686	3,297	0.80 (0.72-0.90)	0.83
40-60	2,920	3,296	1	1	1,835	3,296	1	1	869	3,296	1	1
60-80	3,519	3,297	1.20 (1.12-1.29)	1.23	2,302	3,297	1.24 (1.15-1.34)	1.28	1,061	3,297	1.21 (1.09-1.35)	1.19
80-90	2,223	1,648	1.51 (1.39-1.64)	1.51	1,449	1,648	1.57 (1.43-1.73)	1.59	593	1,648	1.32 (1.17-1.49)	1.44
90-95	1,318	824	1.82 (1.65-2.02)	1.74	869	824	1.93 (1.72-2.16)	1.87	363	824	1.62 (1.39-1.87)	1.64
95-99	1,325	660	2.22 (1.99-2.47)	2.15	871	660	2.28 (2.02-2.57)	2.43	374	660	2.06 (1.77-2.39)	1.94
>99	410	164	2.72 (2.24-3.29)	2.94	265	164	2.84 (2.30-3.49)	3.33	112	164	2.29 (1.77-2.97)	2.66

PRS was categorised into quantiles based on the PRS distribution in controls and the middle quintile was used as the reference category. Observed odds ratios (ORs) were compared with those predicted under a theoretical polygenic model in which the log OR depends-linearly on the PRS.

Supplementary Table 5. Association between standardised 287-SNP PRS and overall breast cancer risk by ethnic subgroups

Percentile (%)	Chinese				Malay				Indian			
	Case, N	Control, N	Estimated OR (95% CI)	Predicted OR	Case, N	Control, N	Estimated OR (95% CI)	Predicted OR	Case, N	Control, N	Estimated OR (95% CI)	Predicted OR
0-20	563	1032	0.58(0.50-0.66)	0.6	110	267	0.49(0.36-0.65)	0.5	57	204	0.49(0.33-0.70)	0.48
20-40	765	1031	0.80(0.70-0.91)	0.8	172	266	0.77(0.60-1.01)	0.77	80	203	0.69(0.48-0.97)	0.68
40-60	955	1031	1	1	222	266	1	1	116	204	1	1
60-80	1205	1031	1.24(1.10-1.40)	1.26	251	266	1.13(0.88-1.45)	1.13	149	203	1.29(0.94-1.76)	1.24
80-90	696	515	1.44(1.25-1.67)	1.46	129	133	1.16(0.86-1.57)	1.16	86	102	1.47(1.02-2.14)	1.46
>90	1052	516	2.19(1.91-2.52)	2.2	200	134	1.79(1.35-2.37)	1.8	92	102	1.57(1.09-2.26)	1.52
Total	5236	5156			1084	1332			580	1018		

Association between standardised 287-SNP polygenic risk scores (PRS) and overall breast cancer risk in Chinese, Malays and Indians: observed and predicted odds ratio by PRS percentile. PRS was categorised into quantiles based on the PRS distribution in controls and the middle quintile was used as the reference category. Observed odds ratios (ORs) for overall breast cancer for women in Malaysian Breast Cancer Genetics (MyBrCa) and Singapore Breast Cancer Cohort (SGBCC) studies were compared with those predicted under a theoretical polygenic model in which the log OR depends-linearly on the PRS.

Supplementary Table 6. SNPs and odd ratios (Ors) for SNPs used in the construction of other PRSs.

SNPs	CHR	POS[1]	Allele[2]	GENE	Low et al.[3]	Lee et al.[3]	Wen et al.[3]	Hsieh et al.[3]	Chen et al.[3,4]	Overlap with PRS287
rs616488	1	10566215	A/G	*PEX14*		0.94	0.93			YES
rs11552449	1	114448389	C/T	*PTPN22/BCL2L15/AP4B1/DCLRE1B/HIPK1*		1.06				
rs11249433	1	121280613	A/G	*EMBP1*		1.09	1.09		0.80	YES
rs12405132	1	145644984	C/T	*RNF115*					0.98	
rs4951011	1	203766331	A/G	*ZC3H11A*			1.06		1.07	
rs12710696	2	19320803	T/C	*MIR4757*			0.95			
rs4849887	2	121245122	T/C	*INHBB-*		1.09	1.07			
rs2016394	2	172972971	G/A	*METAP1D/DLX1/DLX2*		0.95				
rs10931936	2	202143928	T/C	*CASP8*			0.95			
rs13387042	2	217905832	A/G	*TNP1à*		0.93	0.95			
rs16857609	2	218296508	C/T	*DIRC3*		0.93	1.09			
rs6762644	3	4742276	A/G	*ITPR1/EGOT*		1.07				
rs4973768	3	27416013	C/T	*SLC4A7E*		1.11	1.11		1.15	
rs1431131	3	30675880	A/T	*TGFBR2*					1.01	
rs12493607	3	30682939	G/C	*TGFBR2*		0.94	1.05			
rs6796502	3	46866866	G/A	*PRSS42*					1.08	
rs7696175	4	38820986	T/C	*TLR6*					0.93	
rs9790517	4	106084778	C/T	*TET2*		1.05				
rs6828523	4	175846426	C/A	*ADAM29*		0.90	0.94			
rs10069690	5	1279790	C/T	*TERT*			1.07		1.07	YES
rs2242652	5	1280028	G/A	*TERT*					1.06	
rs13162653	5	16187528	G/T	*LOC401176*					0.87	
rs2012709	5	32567732	C/T	*SUB1*					1.05	
rs4415084	5	44662515	C/T	*5p12 (intergenic)*		0.87				
rs10941679	5	44706498	A/G	*MRPS30*		1.12	1.07			YES
rs981782	5	45285718	A/C	*HCN1*				1.29		
rs16886165	5	56023083	T/G	*MAP3K1*					1.21	YES
rs889312	5	56031884	C/A	*MAP3K1*		0.95	0.96	0.76		
rs2229882	5	56168712	C/T	*MAP3K1*					1.32	
rs10472076	5	58184061	T/C	*RAB3C*		1.04				
rs7707921	5	81538046	T/A	*ATG10*					0.85	
rs10474352	5	90732225	C/T	*RAB5CP2*			0.93		1.01	
rs1432679	5	158244083	C/T	*EBF1*		0.93	0.93			YES
rs11242675	6	1318878	C/T	*FOXQ1*		1.15				
rs204247	6	13722523	G/A	*RANBP9*		0.95				
rs17529111	6	82128386	T/C	*FAM46A*		1.06				
rs2180341	6	127600630	G/A	*ECHDC1/RNF146*		0.76				
rs9485372	6	149608874	G/A	*TAB2*			0.88			
rs3757318	6	151914113	G/A	*ESR1*		1.04			1.40	
rs12662670	6	151918856	T/G	*ESR1*					1.42	
rs11155804	6	151946152	T/A	*ESR1*					1.42	
rs2046210	6	151948366	G/A	*ESR1*		1.27	1.27		1.42	
rs6557161	6	151950235	A/G	*ESR1*	1.16					
rs4593472	7	130667121	C/T	*LINC-PINT*					0.97	
rs720475	7	144074929	G/A	*ARHGEF5*		0.94				
rs9693444	8	29509616	A/C	*C8orf75*		0.93	0.93			YES
rs13365225	8	36858483	A/G	*KCNU1*					0.92	YES
rs6472903	8	76230301	G/T	*HNF4G*			1.13		0.90	
rs13267382	8	117209548	A/G	*LINC00536*					1.05	YES
rs13281615	8	128355618	A/G	*8q24*		1.07			0.9	
rs7815245	8	128383597	C/T	*CASC8*					0.89	
rs1562430	8	128387852	T/C	*8q24*		0.87	0.94		0.87	
rs1011970	9	22062134	G/T	*CDKN2A*		1.07	1.07			
rs10759243	9	110306115	C/A	*KLF4*		1.06	1.06			
rs10816625	9	110837073	A/G	*CHCHD4P2*					0.83	YES
rs865686	9	110888478	G/T	*CHCHD4P2*		1.11			0.94	
rs10822013	10	64251977	C/T	*ZNF365*		1.08	1.08	1.05		
rs10509168	10	64257828	T/C	*CNF365*	1.10					
rs10995190	10	64278682	G/A	*ZNF365*		1.06			1.14	
rs704010	10	80841148	T/C	*ZMIZ1*		0.93	0.94		0.86	
rs11199914	10	123093901	C/T	*10q26.12*			0.96			
rs11200014	10	123334930	G/A	*FGFR2*					1.07	
rs2981579	10	123337335	A/G	*FGFR2*			0.86		0.88	
rs2981578	10	123340311	C/T	*FGFR2*	0.81					
rs1219648	10	123346190	A/G	*FGFR2*		1.14			1.13	
rs2981582	10	123352317	A/G	*FGFR2*		0.79		0.79	0.88	
rs3817198	11	1909006	T/C	*LSP1*		1.07				
rs909116	11	1941946	T/C	*LSP1*			0.93		0.84	
rs3903072	11	65583066	G/T	*DKFZp761E198/OVOL1/SNX32/CFL1*		0.95				
rs614367	11	69328764	C/T	*CCND1*			1.28			
rs11820646	11	129461171	T/C	*BARX2*		1.05				
rs7107217	11	129473690	A/C	*BARX2*			1.1			
rs12422552	12	14413931	G/C	*ATF7IP*		1.06	1.06			YES
rs10771399	12	28155080	A/G	*PTHLH*		0.86	0.89		0.9	
rs7297051	12	28174817	C/T	*PTHLH*					0.82	YES
rs17356907	12	96027759	A/G	*NTN4*		0.91	0.94			YES
rs1292011	12	115836522	A/G	*MED13L*			0.89			
rs2236007	14	37132769	G/A	*PAX9/SLC25A21*		0.92	0.93			
rs3784099	14	68749927	G/A	*RAD51B*				0.73		
rs941764	14	91841069	A/G	*CCDC88C*		1.06	1.06			YES
rs11627032	14	93104072	T/C	*RIN3*					1.04	
rs2290203	15	91512067	G/A	*PRC1*			0.92		0.95	
rs3803662	16	52586341	A/G	*LOC643714*	0.83		0.88	0.88		
rs4784227	16	52599188	C/T	*TOX3*		1.23	1.23		1.28	YES
rs12922061	16	52635000	C/T	*TOX3*	1.23					
rs3112612	16	52635164	G/A	*TOX3*		0.88				
rs1421085	16	53800954	T/C	*FTO*					1.02	
rs17817449	16	53813367	T/G	*MIR1972-2/FTO*		0.93				
rs11075995	16	53855291	A/T	*FTO*			0.93		0.91	
rs13329835	16	80650805	A/G	*CDYL2*		1.09				
rs745570	17	77781725	A/G	*CBX8*					1.16	YES
rs527616	18	24337424	C/G	*AQP4à*		0.98	1.04			YES
rs1436904	18	24570667	T/G	*CHST9*		0.96				
rs6507583	18	42399590	A/G	*SETBP1*					1.09	
rs2363956	19	17394124	T/G	*ANKLE1*			0.94			
rs4808801	19	18571141	A/G	*ELL*			0.95			
rs3760982	19	44286513	A/G	*C19orf61:KCNN4:LYPD5:ZNF283*		0.94				
rs2823093	21	16520832	G/A	*NRIP1*		0.92				
rs12628403	22	39358037	A/C	*APOBEC3A*			1.11			
rs6001930	22	40876234	T/C	*MKL1*					1.06	

[1]Build 37 position; [2]Reference/effect allele; [3]Odds ratio for each SNP reported each respectively study; [4]One SNP rs146699004 was not imputed and hence not included in the analyses

Supplementary References:

1. Phuah SY, *et al.* Triple-negative breast cancer and PTEN (phosphatase and tensin homologue) loss are predictors of BRCA1 germline mutations in women with early-onset and familial breast cancer, but not in women with isolated late-onset breast cancer. *Breast cancer research : BCR* **14**, R142, doi:10.1186/bcr3347 (2012).

2. Kawase T, *et al.* FGFR2 intronic polymorphisms interact with reproductive risk factors of breast cancer: results of a case control study in Japan. *International journal of cancer. Journal international du cancer* **125**, 1946-1952, doi:10.1002/ijc.24505 (2009).
3. Hsu HM, *et al.* Breast cancer risk is associated with the genes encoding the DNA double-strand break repair Mre11/Rad50/Nbs1 complex. *Cancer Epidemiol Biomarkers Prev* **16**, 2024-2032, doi:10.1158/1055-9965.EPI-07-0116 (2007).
4. Ding SL, *et al.* Genetic variants of BLM interact with RAD51 to increase breast cancer susceptibility. *Carcinogenesis* **30**, 43-49, doi:10.1093/carcin/bgn233 (2009).
5. Zheng W, *et al.* Genome-wide association study identifies a new breast cancer susceptibility locus at 6q25.1. *Nature genetics* **41**, 324-328, doi:10.1038/ng.318 (2009).
6. Lee KM, *et al.* Genetic polymorphisms of ataxia telangiectasia mutated and breast cancer risk. *Cancer Epidemiol Biomarkers Prev* **14**, 821-825, doi:10.1158/1055-9965.EPI-04-0330 (2005).
7. Han S, *et al.* CASP8 polymorphisms, estrogen and progesterone receptor status, and breast cancer risk. *Breast cancer research and treatment* **110**, 387-393, doi:10.1007/s10549-007-9730-5 (2008).
8. Kwong A, *et al.* Novel BRCA1 and BRCA2 genomic rearrangements in Southern Chinese breast/ovarian cancer patients. *Breast cancer research and treatment* **136**, 931-933, doi:10.1007/s10549-012-2292-1 (2012).
9. Kwong A, *et al.* Identification of BRCA1/2 founder mutations in Southern Chinese breast cancer patients using gene sequencing and high resolution DNA melting analysis. *PloS one* **7**, e43994, doi:10.1371/journal.pone.0043994 (2012).
10. Shimada N, *et al.* Genetic polymorphisms in estrogen metabolism and breast cancer risk in case-control studies in Japanese, Japanese Brazilians and non-Japanese Brazilians. *J Hum Genet* **54**, 209-215, doi:10.1038/jhg.2009.13 (2009).
11. Han SA, *et al.* The Korean Hereditary Breast Cancer (KOHBRA) study: protocols and interim report. *Clin Oncol (R Coll Radiol)* **23**, 434-441, doi:10.1016/j.clon.2010.11.007 (2011).
12. Wu AH, McKean-Cowdin R, Tseng CC. Birth weight and other prenatal factors and risk of breast cancer in Asian-Americans. Breast Cancer Res Treat. 2011;14:917–925.
13. John EM, *et al.* The Breast Cancer Family Registry: an infrastructure for cooperative multinational, interdisciplinary and translational studies of the genetic epidemiology of breast cancer. *Breast cancer research : BCR* **6**, R375-389, doi:10.1186/bcr801 (2004).
14. Grundy A, *et al.* Shift work, circadian gene variants and risk of breast cancer. *Cancer epidemiology* **37**, 606-612, doi:10.1016/j.canep.2013.04.006 (2013).

15. Kobayashi LC, *et al.* Moderate-to-vigorous intensity physical activity across the life course and risk of pre- and post-menopausal breast cancer. *Breast cancer research and treatment* **139**, 851-861, doi:10.1007/s10549-013-2596-9 (2013).
16. Grundy A, *et al.* Increased risk of breast cancer associated with long-term shift work in Canada. *Occupational and environmental medicine* **70**, 831-838, doi:10.1136/oemed-2013-101482 (2013).
17. Kobayashi LC, *et al.* A case-control study of lifetime light intensity physical activity and breast cancer risk. *Cancer causes & control : CCC* **25**, 133-140, doi:10.1007/s10552-013-0312-z (2014).
18. Hankin JH, *et al.* Singapore Chinese Health Study: development, validation, and calibration of the quantitative food frequency questionnaire. Nutr Cancer: 39:187–195 (2001).
19. Wu AH, *et al.* Soy intake and breast cancer risk in Singapore Chinese Health Study. Br J Cancer: 99(1):196-200. doi: 10.1038/sj.bjc.6604448 (2008).

Supplementary Note 1

Supplementary Funding

BCAC is funded by Cancer Research UK [C1287/A16563, C1287/A10118], the European Union's Horizon 2020 Research and Innovation Programme (grant numbers 634935 and 633784 for BRIDGES and B-CAST respectively), and by the European Community´s Seventh Framework Programme under grant agreement number 223175 (grant number HEALTH-F2-2009-223175) (COGS). The EU Horizon 2020 Research and Innovation Programme funding source had no role in study design, data collection, data analysis, data interpretation or writing of the report.

Genotyping of the OncoArray was funded by the NIH Grant U19 CA148065, and Cancer UK Grant C1287/A16563 and the PERSPECTIVE project supported by the Government of Canada through Genome Canada and the Canadian Institutes of Health Research (grant GPH-129344) and, the Ministère de l'Économie, Science et Innovation du Québec through Genome Québec and the PSRSIIRI-701 grant, and the Quebec Breast Cancer Foundation. Funding for the iCOGS infrastructure came from: the European Community's Seventh Framework Programme under grant agreement n° 223175 (HEALTH-F2-2009-223175) (COGS), Cancer Research UK (C1287/A10118, C1287/A10710, C12292/A11174, C1281/A12014, C5047/A8384, C5047/A15007, C5047/A10692, C8197/A16565), the National Institutes of Health (CA128978) and Post-Cancer GWAS initiative (1U19 CA148537, 1U19 CA148065 and 1U19 CA148112 - the GAME-ON initiative), the Department of Defence (W81XWH-10-1-0341), the Canadian Institutes of Health Research (CIHR) for the CIHR Team in Familial Risks of Breast Cancer, and Komen Foundation for the Cure, the Breast Cancer Research Foundation, and the Ovarian Cancer Research Fund. The DRIVE Consortium was funded by U19 CA148065.

The ACP study is funded by the Breast Cancer Research Trust, UK, and, KM and AL are supported by the NIHR Manchester Biomedical Research Centre and by the ICEP ("This work was also supported by CRUK [grant number C18281/A19169]"). The AHS study is supported by the intramural research program of the National Institutes of Health, the National Cancer Institute (grant number Z01-CP010119), and the National Institute of Environmental Health Sciences (grant number Z01-ES049030). CBCS is funded by the Canadian Cancer Society (grant # 313404) and the Canadian Institutes of Health Research. CCGP is supported by funding from the University of Crete. The HERPACC was supported by MEXT Kakenhi (No. 170150181 and 26253041) from the Ministry of Education, Science, Sports, Culture and Technology of Japan, by a Grant-in-Aid for the Third Term Comprehensive 10-Year Strategy for Cancer Control from Ministry Health, Labour and Welfare of Japan, by Health and Labour Sciences Research Grants for Research on Applying Health Technology from Ministry Health, Labour and Welfare of Japan, by National Cancer Center Research and Development Fund, and "Practical Research for Innovative Cancer Control (15ck0106177h0001)" from Japan Agency for Medical Research and development, AMED, and Cancer Bio Bank Aichi. The KOHBRA study was partially supported by a grant from the Korea Health Technology R&D Project through the Korea Health Industry Development Institute (KHIDI), and the National R&D Program for Cancer Control, Ministry of Health & Welfare, Republic of Korea (HI16C1127;

1020350; 1420190). LAABC is supported by grants (1RB-0287, 3PB-0102, 5PB-0018, 10PB-0098) from the California Breast Cancer Research Program. The Northern California Breast Cancer Family Registry (NC-BCFR) and Ontario Familial Breast Cancer Registry (OFBCR) were supported by grant UM1 CA164920 from the National Cancer Institute (USA). The content of this manuscript does not necessarily reflect the views or policies of the National Cancer Institute or any of the collaborating centers in the Breast Cancer Family Registry (BCFR), nor does mention of trade names, commercial products, or organizations imply endorsement by the USA Government or the BCFR. The Carolina Breast Cancer Study was funded by Komen Foundation, the National Cancer Institute (P50 CA058223, U54 CA156733, U01 CA179715), and the North Carolina University Cancer Research Fund. The NGOBCS was supported by the National Cancer Center Research and Development Fund (Japan). The NHS was supported by NIH grants P01 CA87969, UM1 CA186107, and U19 CA148065. The SBCGS was supported primarily by NIH grants R01CA64277, R01CA148667, UMCA182910, and R37CA70867. Biological sample preparation was conducted the Survey and Biospecimen Shared Resource, which is supported by P30 CA68485. The scientific development and funding of this project were, in part, supported by the Genetic Associations and Mechanisms in Oncology (GAME-ON) Network U19 CA148065. SEBCS was supported by the BRL (Basic Research Laboratory) program through the National Research Foundation of Korea funded by the Ministry of Education, Science and Technology (2012-0000347). SGBCC is funded by the NUS start-up Grant, National University Cancer Institute Singapore (NCIS) Centre Grant and the NMRC Clinician Scientist Award. Additional controls were recruited by the Singapore Consortium of Cohort Studies-Multi-ethnic cohort (SCCS-MEC), which was funded by the Biomedical Research Council, grant number: 05/1/21/19/425. The TWBCS is supported by the Taiwan Biobank project of the Institute of Biomedical Sciences, Academia Sinica, Taiwan.

Supplementary Acknowledgements

We thank all the individuals who took part in these studies and all the researchers, clinicians, technicians and administrative staff who have enabled this work to be carried out. The COGS study would not have been possible without the contributions of the following: Per Hall (COGS); Paul Pharoah (BCAC), Andrew Lee, and Ed Dicks, Craig Luccarini and the staff of the Centre for Genetic Epidemiology Laboratory, Javier Benitez, Anna Gonzalez-Neira and the staff of the CNIO genotyping unit, Daniel C. Tessier, Francois Bacot, Daniel Vincent, Sylvie LaBoissière and Frederic Robidoux and the staff of the McGill University and Génome Québec Innovation Centre, Stig E. Bojesen, Sune F. Nielsen, Borge G. Nordestgaard, and the staff of the Copenhagen DNA laboratory, and Julie M. Cunningham, Sharon A. Windebank, Christopher A. Hilker, Jeffrey Meyer and the staff of Mayo Clinic Genotyping Core Facility.

The ACP study wishes to thank the participants in the Thai Breast Cancer study. Special Thanks also go to the Thai Ministry of Public Health (MOPH), doctors and nurses who helped with the data collection process. Finally, the study would like to thank Dr Prat Boonyawongviroj, the former Permanent Secretary of MOPH and Dr Pornthep Siriwanarungsan, the Department Director-General of Disease Control who have supported the study throughout. BBCS thanks Eileen Williams, Elaine Ryder-Mills, Kara

Sargus. CBCS thanks study participants, co-investigators, collaborators and staff of the Canadian Breast Cancer Study, and project coordinators Agnes Lai and Celine Morissette. HKBCS thanks Hong Kong Sanatorium and Hospital, Dr Ellen Li Charitable Foundation, The Kerry Group Kuok Foundation, National Institute of Health 1R03CA130065 and the North California Cancer Center for support. We thank all investigators of the KOHBRA (Korean Hereditary Breast Cancer) Study. LAABC thanks all the study participants and the entire data collection team, especially Annie Fung and June Yashiki. MYBRCA thanks study participants and research staff (particularly Patsy Ng, Nurhidayu Hassan, Yoon Sook-Yee, Daphne Lee, Lee Sheau Yee, Phuah Sze Yee and Norhashimah Hassan) for their contributions and commitment to this study. SBCGS thank study participants and research staff for their contributions and commitment to the studies. SGBCC thanks the participants and research coordinator Ms Tan Siew Li. The authors thank the WHI investigators and staff for their dedication and the study participants for making the program possible.
