# Supplementary material for: European polygenic risk score for prediction of breast cancer shows similar performance in Asian women
Source: Nat Commun. 2020 Jul 31;11:3833. doi: 10.1038/s41467-020-17680-w (PMC7395776; doi:10.1038/s41467-020-17680-w)
Supplement: Supplementary file 3 — Description of Additional Supplementary Files [file 41467_2020_17680_MOESM3_ESM.pdf]

Description of Additional Supplementary Files

File Name: Supplementary Data 1
Description: SNPs and odd ratios (Ors) for 313 SNPs used in the construction of overall breast cancer and subtype-Specific PRSs.
