# Supplementary material for: European polygenic risk score for prediction of breast cancer shows similar performance in Asian women
Source: Nat Commun. 2020 Jul 31;11:3833. doi: 10.1038/s41467-020-17680-w (PMC7395776; doi:10.1038/s41467-020-17680-w)
Supplement: Supplementary file 5 — Reporting Summary [file 41467_2020_17680_MOESM5_ESM.pdf]

nature research

Corresponding author(s): Weang-Kee Ho, Soo-Hwang Teo

Last updated by author(s): Jun 26, 2020

Reporting Summary

Nature Research wishes to improve the reproducibility of the work that we publish. This form provides structure for consistency and transparency in reporting. For further information on Nature Research policies, see Authors & Referees and the Editorial Policy Checklist.

Statistics

For all statistical analyses, confirm that the following items are present in the figure legend, table legend, main text, or Methods section.

n/a	Confirmed	
☐	☒	The exact sample size (*n*) for each experimental group/condition, given as a discrete number and unit of measurement
☒	☐	A statement on whether measurements were taken from distinct samples or whether the same sample was measured repeatedly
☐	☒	The statistical test(s) used AND whether they are one- or two-sided<br>*Only common tests should be described solely by name; describe more complex techniques in the Methods section.*
☐	☒	A description of all covariates tested
☐	☒	A description of any assumptions or corrections, such as tests of normality and adjustment for multiple comparisons
☐	☒	A full description of the statistical parameters including central tendency (e.g. means) or other basic estimates (e.g. regression coefficient) AND variation (e.g. standard deviation) or associated estimates of uncertainty (e.g. confidence intervals)
☐	☒	For null hypothesis testing, the test statistic (e.g. *F*, *t*, *r*) with confidence intervals, effect sizes, degrees of freedom and *P* value noted<br>*Give P values as exact values whenever suitable.*
☒	☐	For Bayesian analysis, information on the choice of priors and Markov chain Monte Carlo settings
☒	☐	For hierarchical and complex designs, identification of the appropriate level for tests and full reporting of outcomes
☐	☒	Estimates of effect sizes (e.g. Cohen's *d*, Pearson's *r*), indicating how they were calculated

Our web collection on statistics for biologists contains articles on many of the points above.

Software and code

Policy information about availability of computer code

Data collection	Data were contributed by participating studies and centrally harmonised and managed by Breast Cancer Association Consortium
Data analysis	All statistical analysis in this study were conducted using R v.3.0.3 or Strata v.14.2. Genotyping and Imputation were done as part of previous studies where SHAPEIT version 2 and IMPUTE version 2 were used for phasing and imputation, respectively.

For manuscripts utilizing custom algorithms or software that are central to the research but not yet described in published literature, software must be made available to editors/reviewers. We strongly encourage code deposition in a community repository (e.g. GitHub). See the Nature Research guidelines for submitting code & software for further information.

Data

Policy information about availability of data

All manuscripts must include a data availability statement. This statement should provide the following information, where applicable:

- Accession codes, unique identifiers, or web links for publicly available datasets
- A list of figures that have associated raw data
- A description of any restrictions on data availability

Summary statistics (odds ratios and confidence limits) for all SNPs included in this study are provided in Supplementary Table 2 of the manuscript. Individual patient data cannot be made publicly available due to restraints imposed by ethic committees of individual studies and should be requested via the Data Access Coordination Committee of BCAC (BCAC Coordinator: BCAC@medschl.cam.ac.uk). Source data of figures are provided with this paper.

Field-specific reporting

Please select the one below that is the best fit for your research. If you are not sure, read the appropriate sections before making your selection.

☒ Life sciences ☐ Behavioural & social sciences ☐ Ecological, evolutionary & environmental sciences

For a reference copy of the document with all sections, see nature.com/documents/nr-reporting-summary-flat.pdf

Life sciences study design

All studies must disclose on these points even when the disclosure is negative.

Sample size

This study include 45,233 women of Asian ancestry. To our knowledge, this is the largest study of Asian women to date on which the PRS could be evaluated; therefore, the power was essentially limited by the available dataset. When the polygenic risk score (PRS) was treated as continuous variable, a total of 691 cases and 691 controls are required to achieve 80% power to detect odds ratio (OR) of 1.33 (the lowest value observed in our analysis for continuous PRS) at 0.001% significance level. When PRS was treated as categorical variable, to achieve 80% power to detect OR of 2.7 (top 1% of PRS distribution versus middle quintile) at 0.001% significance level, a total sample size of 3,855 cases and matching controls are needed. Thus, our study was very well powered for both types of analysis (PRS treated as continuous/categorical variable) except for the ER-subtype analysis in Asian American and ethnic specific analysis, where the proportion of ER-negative disease is limited. We have noted in the manuscript that sample size is a limitation for ER-specific analyses. It should also be noted that the aim of this study was to estimate the effect size the PRS in Asian women, therefore the precision of the estimates is more relevant than the power.

Data exclusions

Using the pre-established QC criteria from Breast Cancer Association Consortium, we filtered samples found to be genotypically not female, discordant or cryptic duplicate pairs, and samples with assay call rate <95% and extreme heterozygosity. For the first-degree relative pairs, the control was removed from the case-control pairs; otherwise the samples with lower call rate was excluded. Before combining common genetic variants as reported in the literature into polygenic risk scores, we also excluded genetic variants that have low imputation quality score.

Replication

In common with most large-scale genotyping experiments, samples were genotyped once. Array-based genotyping in general is highly accurate, and we have shown previously that the Oncoarray in particular gives highly reproducible genotypes between replicate samples.

Randomization

Our study did not involve randomisation of samples into experimental groups. Participants were classified based on their breast cancer status. We included principle components in the association analyses to adjust for population structure.

Blinding

Our study did not involve randomisation of samples into experimental groups hence blinding was not relevant to our study

Reporting for specific materials, systems and methods

We require information from authors about some types of materials, experimental systems and methods used in many studies. Here, indicate whether each material, system or method listed is relevant to your study. If you are not sure if a list item applies to your research, read the appropriate section before selecting a response.

Materials & experimental systems

n/a	Involved in the study
☒	☐ Antibodies
☒	☐ Eukaryotic cell lines
☒	☐ Palaeontology
☒	☐ Animals and other organisms
☐	☒ Human research participants
☒	☐ Clinical data

Methods

n/a	Involved in the study
☒	☐ ChIP-seq
☒	☐ Flow cytometry
☒	☐ MRI-based neuroimaging

Human research participants

Policy information about studies involving human research participants

Population characteristics

Participants were Asian women recruited into 14 participating studies, of which ~39% were diagnosed with breast cancer. Of the 14 studies, participants of 11 studies were recruited in Asia while participants of the remaining 3 studies were from North America. All subjects were of Asian ancestry, as defined by principal components analysis. The overall mean age of controls and mean age of diagnosis from the case from the case-control studies are 50.6 and 50.7, respectively. The mean age of controls ranging from 41.35 to 57.05 for individual study while the mean age of diagnosis ranging from 39.98 to 55.29.

Recruitment

Participants were recruited by following the recruitment protocol of individual study. We have included the study design of each study in Supplementary Table 1. The main analyses are very unlikely to be affected by self-selection biases, as the subjects will not have been aware of their genotype and the genotyping is highly accurate. The family history data were, however, self-

reported and many have been subject to recall bias. We showed that there was evidence of heterogeneity in the effect sizes of association between family history and breast cancer risk across studies, and have noted this as a limitation in the manuscript.

Ethics oversight	All studies were approved by the relavant institutional ethics committees and review boards, and all the participants provided written informed consent.

Note that full information on the approval of the study protocol must also be provided in the manuscript.
